# Conserved N- and C-terminal motifs of PAD-1 are required to inhibit extracellular vesicle release

**DOI:** 10.17912/micropub.biology.000625

**Published:** 2022-09-14

**Authors:** Lauren R Pitts, Alexander T Nguyen, Ann M Wehman

**Affiliations:** 1 Department of Biological Sciences, University of Denver, Denver, CO, USA

## Abstract

Cells release extracellular vesicles (EVs) carrying cargos that can influence development and disease, but the mechanisms that govern EV release by plasma membrane budding are poorly understood. We previously showed that the Dopey protein PAD-1 inhibits EV release from the plasma membrane in *C. elegans* . However, PAD-1 is large, and the domains required to regulate EV release were unknown. Here, we reveal that the conserved N-terminal EWAD motif and C-terminal leucine zippers are required to inhibit EV release from the plasma membrane. Revealing a role for these domains is an important first step to identifying how EV release is regulated.

**Figure 1. Mutations in the predicted KLC2-binding and leucine zipper domains of PAD-1 result in a severe increase in extracellular vesicle (EV) release.**
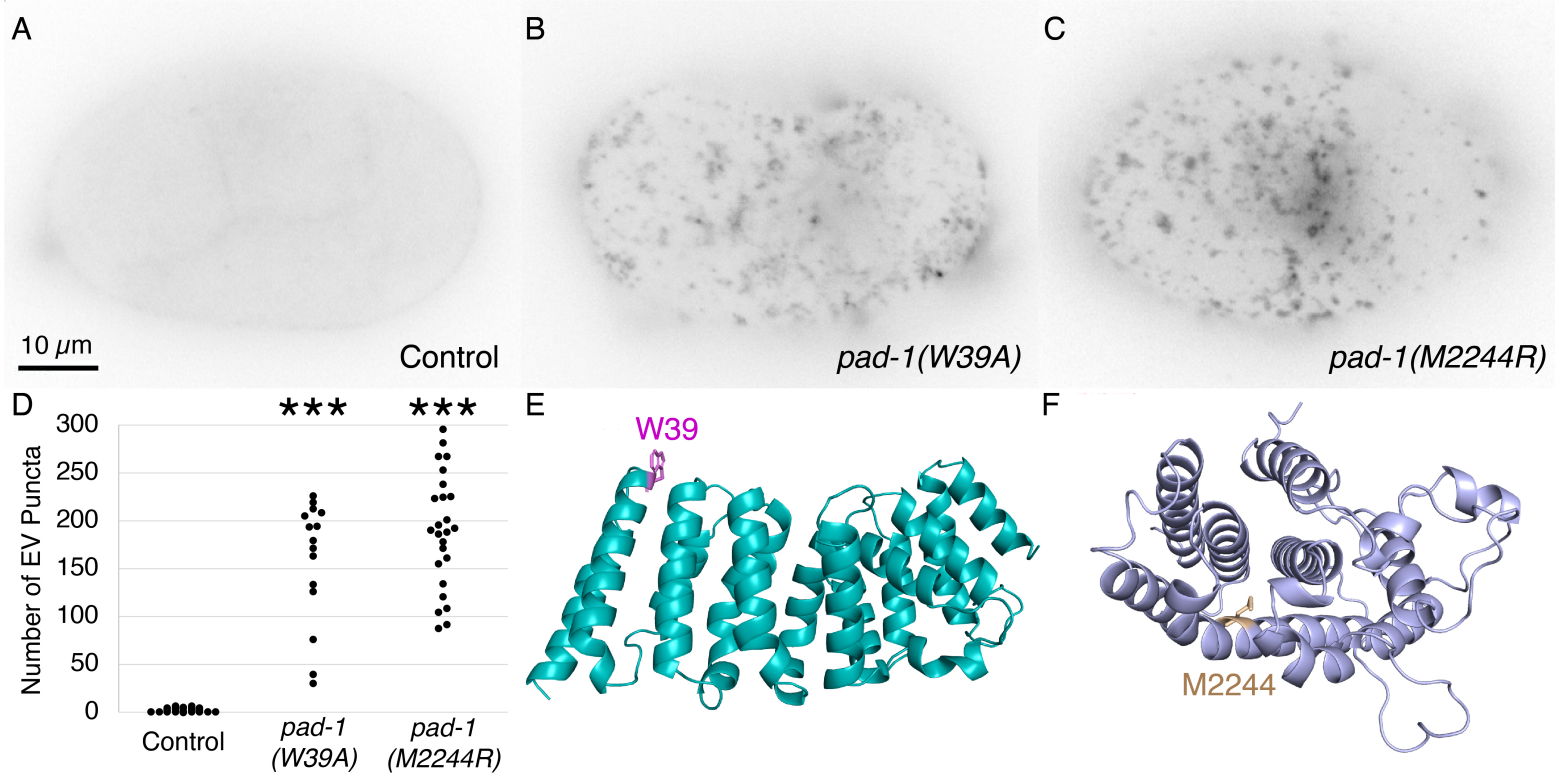
(A-C) Inverted images of the surface of 4-cell embryos expressing mCh::PH::CTPD in control (A), *pad-1(W39A)* KLC2 binding site mutant (B), and *pad-1(M2244R)* leucine zipper mutant (C). (D) The number of mCh::PH::CTPD puncta on the surface of individual *pad-1(W39A)* (n=15) and *pad-1(M2244R)* mutant embryos (n=25) were significantly increased compared to control embryos (n=22, ***p<0.001). (E) AlphaFold2 prediction of N-terminal Dopey domain (aa16-301, W39 magenta). (F) AlphaFold2 prediction of C-terminal domain (aa2165-2417, M2244 beige).

## Description

The Dopey family protein PAD-1 is a key regulator of extracellular vesicle (EV) release in *Caenorhabditis elegans* (Beer et al., 2018; Fazeli et al., 2020). PAD-1 is thought to inhibit EV release by activating the phospholipid flippase TAT-5 to maintain phosphatidylethanolamine (PE) asymmetry in the plasma membrane. When PAD-1 is disrupted, cytofacial PE is externalized, membrane-sculpting ESCRT complexes are recruited to the plasma membrane, and EVs bud from the plasma membrane by ectocytosis (Beer et al., 2018; Beer, 2021). However, which domains of PAD-1 are important to regulate EV release was unknown.

PAD-1 has a conserved N-terminal Dopey domain and C-terminal leucine zipper-like domain, similar to other large Dopey proteins (Molière et al., 2022). In mammals, the N-terminal Dopey domain binds to the kinesin light chain KLC2 via a tryptophan surrounded by acidic residues (EWAD motif) (Mahajan et al., 2019), which is proposed to link Kinesin-1 to membranes for organelle trafficking (Mahajan et al., 2019; Zhao et al., 2020). Indeed, an LKRL motif in the C-terminal leucine zipper region of Dopey1 has been shown to bind lipids (Mahajan et al., 2019). Furthermore, the leucine zippers play essential roles in Dopey protein function, as a mutation in the leucine zipper domain of the Dopey protein DopA (I1695R) disrupts cellular morphogenesis in *Aspergillus nidulans* (Pascon & Miller, 2000). Leucine zippers are often involved in protein-protein interactions (Landschulz et al., 1988), but it is unknown whether specific proteins interact with this domain. Furthermore, whether the N-terminal EWAD motif or C-terminal leucine zippers are required to inhibit EV release was unknown.

To determine the role of the N-terminal Dopey domain in EV release, we used CRISPR/Cas9-mediated genome editing to mutate tryptophan 39 to alanine (W39A) in the EWAD motif of PAD-1 (Fig. 1E). We found that *pad-1(W39A)* mutants were mostly sterile and that their few embryos were embryonic lethal, similar to *pad-1* deletion mutants (Beer et al., 2018). To test for an increase in EV release, we crossed the *pad-1(W39A)* mutants with a degron-tagged plasma membrane reporter, mCh::PH::CTPD. The CTPD degron targets proteins for degradation in the cytosol starting at the first mitotic division, allowing us to specifically label EVs released from the plasma membrane starting after the 2-cell stage (Beer et al., 2019). We observed a 50-fold increase in EV puncta on the surface of *pad-1(W39A)* mutant embryos compared to control embryos (Fig. 1A-B, D). These results demonstrate that the N-terminal EWAD motif is required to inhibit EV release, suggesting that PAD-1 binding to Kinesin-1 or other proteins through its Dopey domain regulates EV release.

To test a role for the C-terminal leucine zippers in EV release, we next created a PAD-1(M2244R) allele (Fig. 1F), which corresponds to the DopA I1695R mutant in *Aspergillus* (Pascon & Miller, 2000), and an LKRL motif mutant. We discovered that *pad-1(M2244R)* mutants were mostly sterile and that their few embryos were embryonic lethal, similar to *pad-1(W39A)* and deletion mutants (Beer et al., 2018). In contrast, *pad-1(LKRL-AAAA)* mutants were entirely sterile, precluding the investigation of embryonic EVs. After crossing the mCh::PH::CTPD EV reporter with the *pad-1(M2244R)* mutants, we found that the M2244R point mutation resulted in a >60-fold increase in EV puncta (Fig. 1A, C-D). These results suggest that the C-terminal leucine zippers of PAD-1 are crucial for inhibiting EV release by ectocytosis.

By characterizing the effects of point mutations in PAD-1, we demonstrate that both the conserved N-terminal EWAD motif in the Dopey domain and C-terminal leucine zippers of PAD-1 are required to inhibit EV release from the plasma membrane. Although a mammalian Dopey domain binds KLC2 *in vitro* (Mahajan et al., 2019), it remains unclear whether this is the only binding partner of the highly conserved EWAD motif. Furthermore, it will be important to test whether KLC2 plays a role in EV regulation as part of Kinesin-1. It is also unclear what molecules interact with the conserved C-terminal leucine zipper domain of PAD-1, although this region is important for membrane binding (Mahajan et al., 2019). Further investigation into the molecular interactions of the N- and C-terminal PAD-1 domains will help us understand the mechanisms that govern EV release as well as Dopey protein function.

## Methods

**Worm Strains and Maintenance**

*C. elegans* strains were maintained and crossed on Nematode Growth Media (NGM) seeded with OP50 bacteria and grown at room temperature using standard protocols (Brenner 1974). See Table 1 for a list of strains used in this study.

**Table 1: Worm strains**

**Table:** 

**Strain**	**Genotype**	**Source**
N2	Wild Type	Brenner, 1974
FT207	*tat-5(tm1741) I / hT2[bli-4(e937) let-?(q782) qIs48] (I; III)*	Wehman et al., Curr Biol 2011
FX30208	*tmC27[unc-75(tmIs1239[myo-2p::Venus])] I*	Dejima K, et al. Cell Rep. 2018
PHX1681	*pad-1(syb1647[M2244R]) / + I*	SunyBiotech
PHX2032	*pad-1(syb2032[W39A]) / + I*	SunyBiotech
PHX2521	*pad-1(syb2521[LKRLmut]) / tmC27[unc-75(tmIs1239[myo-2p::Venus])] I*	SunyBiotech
WEH434	*unc-119(ed3) III; wurIs155[pAZ132-coPH-oma-1(219-378): pie-1::mCh::coPH::CTPD; unc-119(+)]*	Beer et al., Nat. Commun 2019
WEH490	*pad-1(syb1647[M2244R]) / hT2[bli-4(e937) let-?(q782) qIs48] I; + / hT2 III*	Crossed N2 to FT207, then PHX1681
WEH493	*pad-1(syb1647[M2244R]) / tmC27[unc-75(tmIs1239[myo-2p::Venus])] I*	Crossed N2 to WEH490, then FX30208
WEH516	*pad-1(syb2032[W39A]) / tmC27[unc-75(tmIs1239[myo-2p::Venus])] I*	Crossed N2 to FX30208, then PHX2032
WEH659	*pad-1(syb1647[M2244R]) / tmC27[unc-75(tmIs1239[myo-2p::Venus])] I; unc-119(ed3) III; wurIs155[pAZ132-coPH-oma-1(219-378): pie-1::mCh::coPH::CTPD; unc-119(+)]*	Crossed N2 to WEH493, then WEH434
WEH660	*pad-1(syb2032[W39A]) / tmC27[unc-75(tmIs1239[myo-2p::Venus])] I; unc-119(ed3)? III; wurIs155[pAZ132-coPH-oma-1(219-378): pie-1::mCh::coPH::CTPD; unc-119(+)]*	Crossed N2 to WEH516, then WEH434
WEH661	*pad-1(syb2521[LKRLmut]) / tmC27[unc-75(tmIs1239[myo-2p::Venus])] I; unc-119(ed3)? III; wurIs155[pAZ132-coPH-oma-1(219-378): pie-1::mCh::coPH::CTPD; unc-119(+)]*	Crossed N2 to PHX2521, then WEH434.

**Genome Editing**

The W39A, M2244R, and LKRL mutants were created by SunyBiotech using CRISPR-Cas9-mediated genome editing (Paix et al. 2014). *pad-1(syb2032[W39A])* was generated using the guide RNA ATTCGAAACACCCAATGAATGGG and the repair template ACGCAAAAGCCATCGATCAGGCGTTGAAAACATTCGAAACACCCAATGAA **GCC** GC **G** GATCTCATTTCGGCACTCGGAAAATTGGCTAAAGTGGGTTTTGTTAG.

*pad-1(syb1647[M2244R])* was generated using the guide RNA GATTGGTGTGTGGCCTATTATGG and the repair template GTACTTCTTCTCCGACTCCGCCCACACAGTTTGATTGGTGTGTGGCCTAT **ACGC** GTTACAGAGCTCGTTCACGCACTATCACAGCTTGAACAACAATTACAAAG.

*pad-1(syb2521[LKRLmut])* was generated using the repair template

TCAATTATGACATCAAAAGAGCAAGAATACGAAGCACGTGCTCAAGCA **GCCGCAGCAGCT** ACTTTTGTCGTTTTTGGTAGTCAATTAGATCAATATCACGGGCAGATGAA. Mutations are underlined and in bold.

**Light Microscopy**

Fluorescence images were taken with a Zeiss Axio Observer 7 inverted microscope with a Plan-Apo 40X 1.4 NA oil objective with Excelitas Technologies X-Cite 120LED Boost illumination, and a Hamamatsu ORCA-Fusion sCMOS camera controlled by 3i SlideBook6 software over multiple days (control: 2, W39A: 7, M2244R: 9).

**Image Manipulation**

Images were rotated, cropped, and inverted for clarity, and the intensity was adjusted using Adobe Photoshop 2022. AlphaFold2 models (Jumper et al., 2021) were colorized and segmented in PyMol 2.3.2 (Schrödinger, Inc.).

**EV Counts**

mCh::PH::CTPD puncta were marked and counted on the top surface of 3- to 15-cell embryos using the ImageJ Cell Counter function (FIJI 2.3.0). The number of fluorescent puncta in clusters were estimated according to the average size of discrete mCh::PH::CTPD puncta.

**Statistics**

Statistical significance was tested using Student’s one-tailed t-test with Bonferroni correction to adjust for multiple comparisons.
